# Letter from the New Editor

**DOI:** 10.5334/pb.1305

**Published:** 2024-03-19

**Authors:** Ruth M. Krebs

**Affiliations:** 1 Department of Experimental Psychology, Faculty of Psychology and Educational Sciences, Ghent University, Belgium

**Keywords:** editorial team, PhD critical review, special collections, open access, societal challenges

With great enthusiasm I have joined the editorial team of Psychologica Belgica, the journal of the Belgian Association of Psychological Sciences (BAPS), as new Editor-in-Chief in October 2023 (https://psychologicabelgica.com/about/editorialteam). First, I would like to express my gratitude to the previous Editor-in-Chief Steve Majerus. He has led the journal over two editorial terms from 2018 onwards, including navigating through the COVID-19 pandemic, which undoubtedly put additional strain on the editors, reviewers, and authors. And apart from leaving the journal in excellent shape, Steve was also tremendously supportive towards me and has helped me to get acquainted with the new role and the associated processes.

I would also like to extend a warm welcome to the new members of the editorial team, i.e., Olivier Desmedt, Olivier Luminet, Sabine Pohl, Lara Stas, Anouk Vanden Bogaerde, Andreas von Leupoldt, as well as my appreciation towards all editors who are staying on for a new term (Lieven Brebels, Marc Brysbaert, Jan De Houwer, Bert de Smedt, Denis Drieghe, Nicolas Gillet, Mandy Rossignol, Alain Van Hiel). Finally, I would like to thank the editors whose term ended recently for their continuous selfless efforts in handling an increasing number of submissions (Fabienne Chetail, Wim Gevers, Joeri Hofmans, Etienne Quertemont, Filip Raes, Isabelle Roskam, Florence Stinglhamber, Hervé Tissot).

Over the past three years, Psychologica Belgica has maintained a robust and healthy presence in the field of psychological research. The quality of published articles, with a wide spread of topics and perspectives, showcase the scientific discourse within our community. The journal’s CiteScore and Impact Factor has been continuing to increase over the past years, as have the views and downloads. Looking ahead, we continue to promote a diverse range of contributions that adhere to rigorous experimental practices and embrace open-science principles. In addition to original research articles, short reports, and theoretical reviews, we welcome the submission of PhD critical review papers, providing an avenue for early-career researchers to showcase their work (e.g., Eben, Billieux, & Verbruggen, 2020). Further, Special Collections have become a hallmark of our journal, thanks to the enormous efforts of the guest editors. The current call for papers on the Foundations and advances in statistical methods edited by Marc Brysbaert and Etienne Quertemont is still open (https://psychologicabelgica.com/announcements#call-for-a-special-issue-on-foundations-and-advances-in-statistical-methods). I would like to highlight that all articles submitted to Psychologica Belgica undergo rigorous peer-review, and that we follow a truly open-access approach, which includes open access to the articles and the anonymized data, as well as fair publication fees with a discount for BAPS members.

In line with ongoing debates and initiatives on a national and global level, we recognize the urgency of addressing issues related to climate change, social inequality, and diversity – and the potential contribution of psychological sciences in mitigating these challenges (see, Easterbrook & Hadden, 2021; Nielsen et al., 2021). In keeping with this notion, Psychologica Belgica invites submissions with an applied angle on pressing societal issues grounded in fundamental research. In this vein, we are planning to launch a Special Collection focused on contributions from psychological science to understand and mitigate the climate crisis.

To conclude, I look forward to working together with the editorial team, the authors, and the reviewers to maintain the journal’s high standards and promote contributions to fundamental as well as applied psychological research in the context of contemporary challenges.
